# Supplementary material for: Transcriptome Analysis of Rice Embryo and Endosperm during Seed Germination
Source: Int J Mol Sci. 2023 May 13;24(10):8710. doi: 10.3390/ijms24108710 (PMC10218590; doi:10.3390/ijms24108710)
Supplement: Supplementary file 1 [file ijms-24-08710-s001.zip › Figures.pdf]

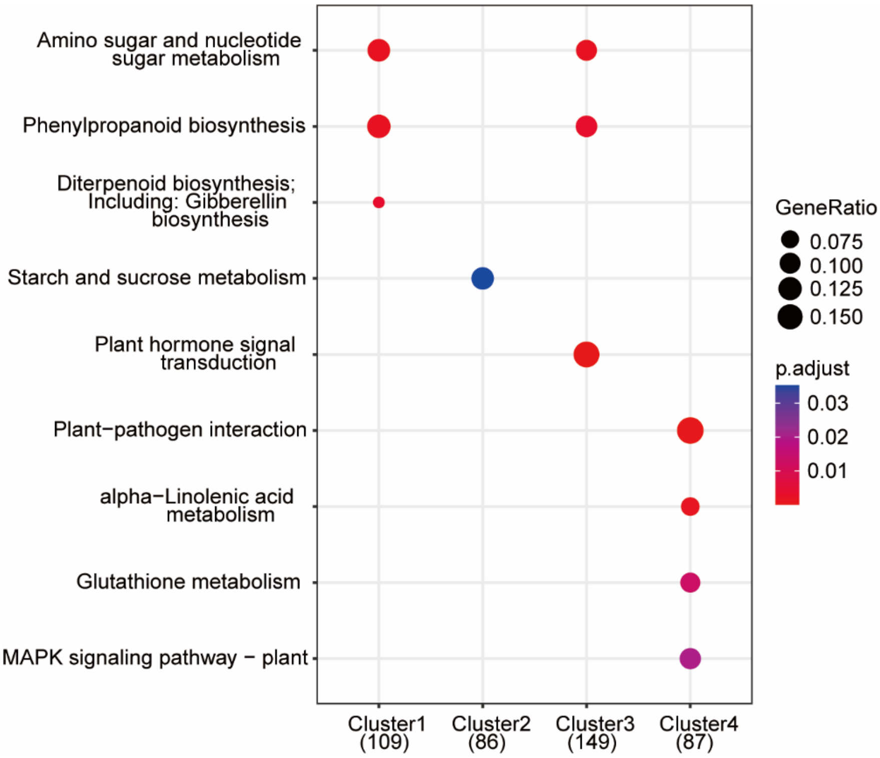

Figure S1. KEGG analysis of consistently differentially expressed genes.

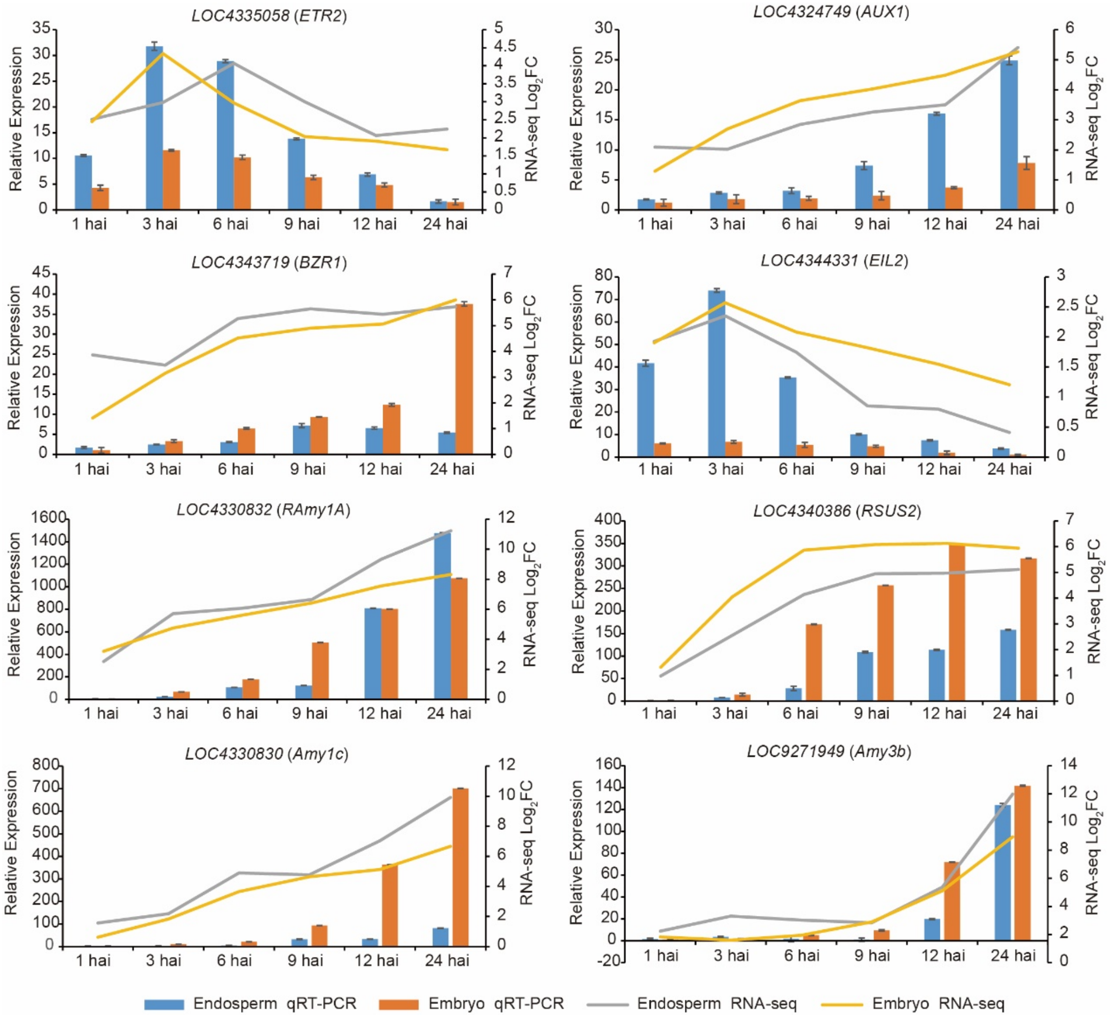

Figure S2. Quantitative RT-PCR validation of 8 DEGs. Expression of DEGs was normalized to the endogenous control *UBQ10* gene. The histogram represents qRT-PCR data of genes expression, with blue represents endosperm and orange represents embryo. The line chart represents Log_2 fold change expression of the DEGs from RNA-seq data, with grey represents endosperm and yellow represents embryo. Three biological replicates of each sample were used for qRT-PCR analysis. Error bars represent the standard deviation of relative expression level from three biological replicates.
